# Supplementary material for: Low T Cell Responsiveness in the Early Phase of COVID-19 Associates with Progression to Severe Pneumonia in Kidney Transplant Recipients
Source: Viruses. 2022 Mar 5;14(3):542. doi: 10.3390/v14030542 (PMC8949290; doi:10.3390/v14030542)
Supplement: Supplementary file 1 [file viruses-14-00542-s001.zip › viruses-1585296-supplementary.pdf]

SUPPLEMENTAL DATA

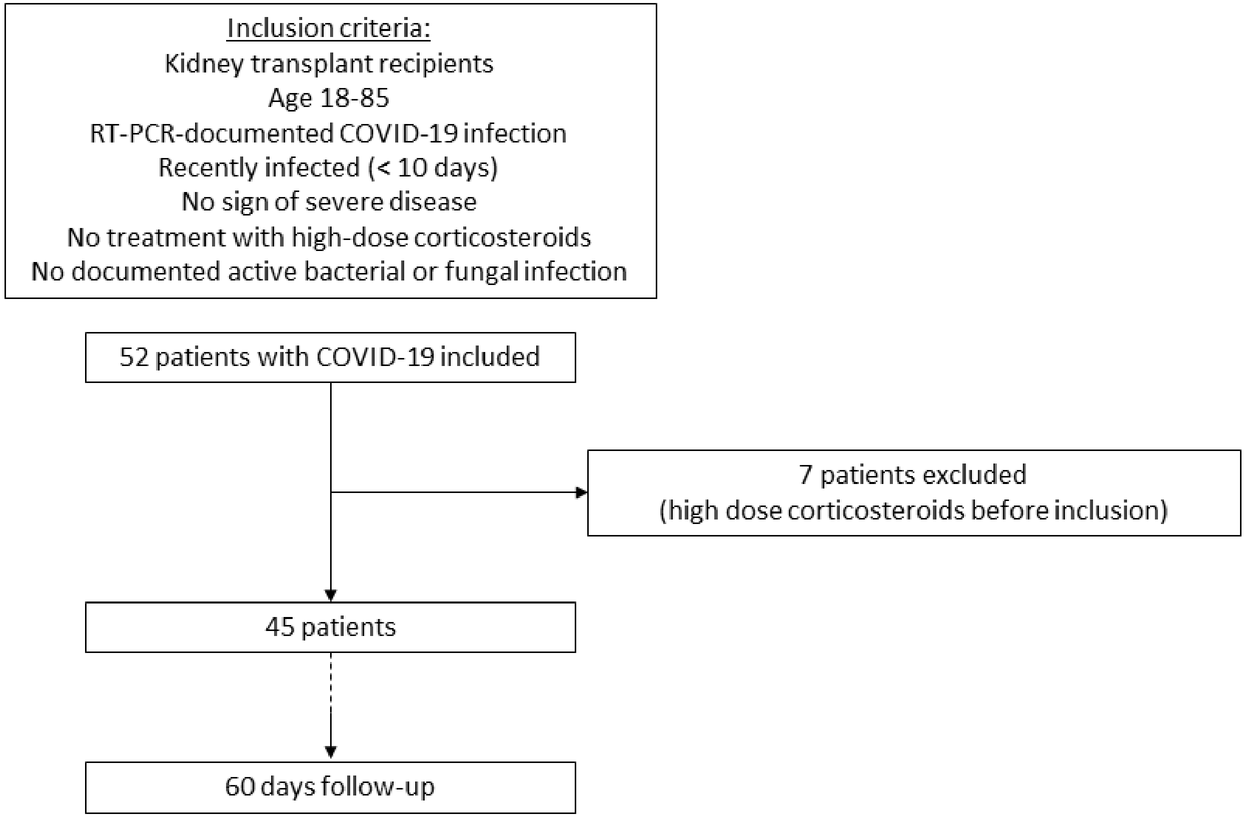

Supplemental Figure S1: Flowchart

Fifty-two KT patients infected with COVID-19 were initially included. Seven patients were excluded from the study because they had received high-dose corticosteroids before blood collection.

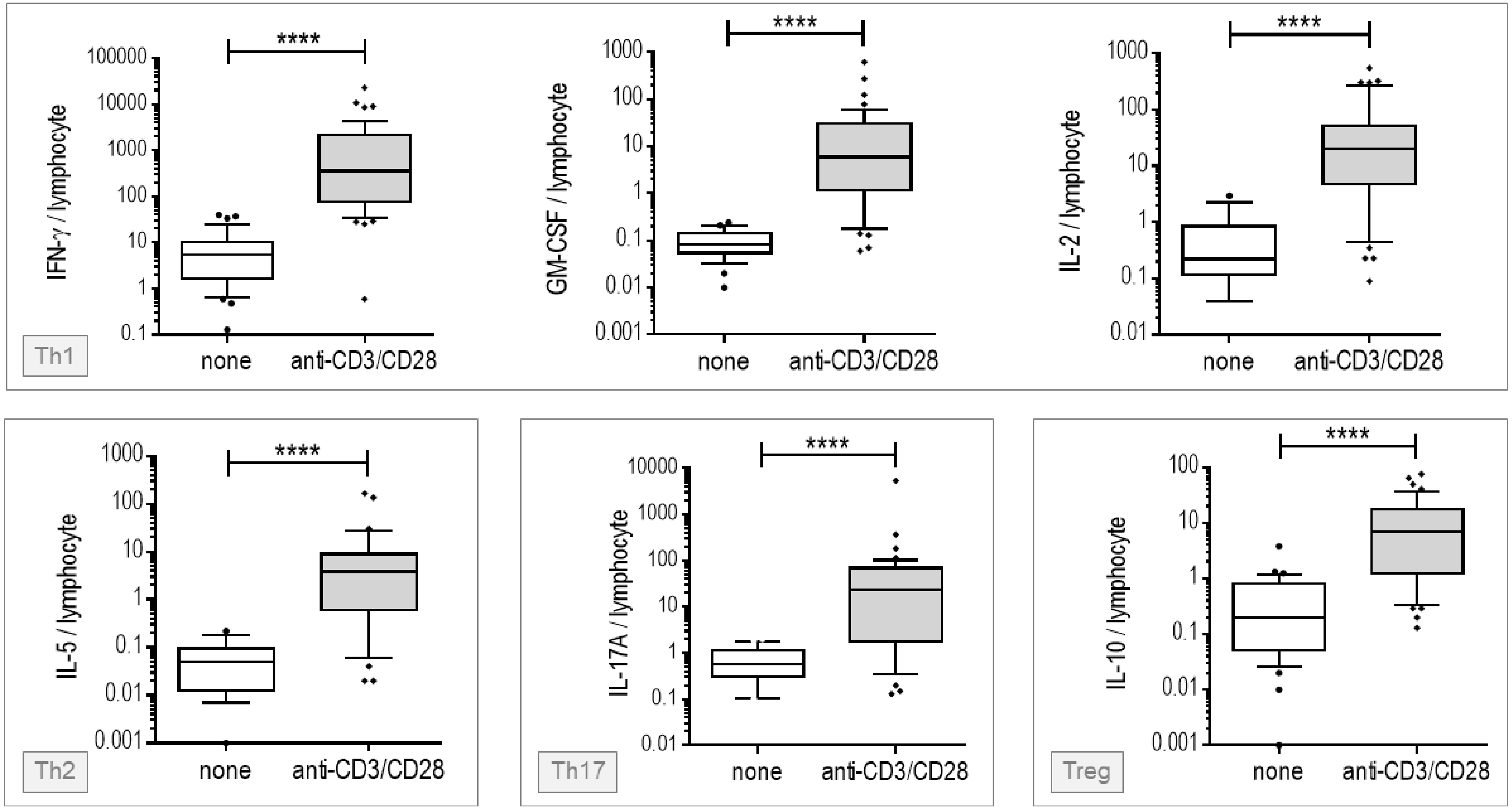

Supplemental Figure S2: Cytokine levels secreted by blood cells.

Blood cells were stimulated at 37°C for 25 hours with or without anti-CD3/CD28 agonist mAbs, and IL-2, IL-5, IL-10, IL-17A, IFN-γ and GM-GSF concentrations were measured in cellular supernatants. Data are presented as box plots. Statistical significance of differences between groups were assessed using the Mann-Whitney non-parametric test.

****, $p < 0.0001$
